# Editorial: Strained Aza-Heterocycles in Synthesis

**DOI:** 10.3389/fchem.2019.00669

**Published:** 2019-10-09

**Authors:** Hyun-Joon Ha

**Affiliations:** Department of Chemistry, Hankuk University of Foreign Studies, Yongin, South Korea

**Keywords:** ring strain, aza-heterocycles, aziridines, azetidines, stereoselective

“Strain” in organic molecules is a powerful driving force able to promote reactivity through “strain-release.” This concept of strain is often wrongly associated with the idea of instability and cumbersome synthetic routes to access strained molecules, but kinetic and thermodynamic worlds ignore each other since strained molecules can be perfectly stable while highly reactive in a “designed” reaction (Couty, 2009; Sweeney, 2009). On the other hand, aza-heterocycles with a prefix of “aza” indicating nitrogen (i.e., nitrogen-containing heterocycles) are ubiquitous and can be found in almost 60% of the 200 top-selling drugs, so that new synthetic tools for their preparation is a hot topic, and source of continuous innovation.

The Topic “strained aza-heterocycles in synthesis” aims at reporting the use of three and four-membered aziridines, azetidines, azetidinones as well as their activated forms (aziridinium and azetidiniums), or their unsaturated forms (azirines and azetines) in synthesis. The inherent strain in these species (whose list is not restricted to cited ones) is the driving force to promote various new synthetic methodologies, such as ring expansions, ring opening, domino processes, etc. that have gained increasing attention over past years, and some have become powerful tools that can be used as valuable building blocks or auxiliaries in total synthesis in elegant key-steps or as innovative tools in diversity-oriented-synthesis. Papers reporting such methodologies, as well as new and optimized synthetic routes to these strained heterocycles were reported with a paper analyzing fundamental aspects of the mechanism of the highly diverse reactions involved in strain-release (D'hooghe and Ha, 2016).

Within this context three papers have been published, two of which are building “strained” aza-heterocycles including 2-acylazetidines and 3-substituted 4-CF_3_-β-lactams by functional group transformation and alkylation. These two papers are featuring how we are able to handle the strained azetidines introducing valuable substituents at the specific position. More specifically, stereo- and enantioselective addition of organolithiums to 2-oxazolinylazetidines followed by acidic hydrolysis of the resulting oxazolidine intermediates yielded 2-acylazetidines. This study done by Musci et al. revealed an unusual reactivity of the C = N bond of the oxazoline group when reacted with an organolithiums in a non-polar solvent such as toluene. The stereochemical aspect of the addition to the C = N double bond was also identified considering the role of the nitrogen lone pair of the azetidine ring as well as of the oxazolinyl group in promoting a complexation to the organolithium reagents. In addition, the report titled “Direct Access to Substituted 4-CF_3_-β-Lactams” was also published by Skibinska et al.. Although fluorine-containing compounds have been widely used in the field of medicinal chemistry due to their pharmacological properties, 4-CF_3_ monobactams functionalized at C-3 are slightly exploited. Mono- and disubstituted 4-CF_3_-β-lactams at the C-3 position have been obtained stereoselectively under basic conditions. A wide range of functionalities such as alcohols, alkyls, aryls, esters, and double and triple bonds have been introduced. The other is my own groups' paper dealing with the aza-ring transformation by aziridine ring-opening with release of strain associated with the nitrogen-containing three membered ring. Alkylative ring-opening of bicyclic aziridinium ion generated from 4-hydroxybutylaziridine with organocopper reagent was successfully achieved to afford 2-alkylsubstituted piperidine in high or moderate yield. This method allowed a new carbon-carbon bond formation of “non-activated” aziridine via ring-opening of aziridinium ion in regio- and stereo-selective manner. This newly developed reaction was applied for efficient synthesis of alkaloids with the representative examples of natural products including conine and epiquinamide.

As I previously mentioned, the chemistry related to the stereo and regioselective synthesis of strained aza-heterocycles bearing various substituents at specific positions, as well as their transformations, is still challenging. Under this circumstance, extensive studies on strained aza-heterocycles are still on demand in order to develop more efficient synthetic methods.

## Author Contributions

The author confirms being the sole contributor of this work and has approved it for publication.

### Conflict of Interest

The author declares that the research was conducted in the absence of any commercial or financial relationships that could be construed as a potential conflict of interest.

## References

[B1] Couty F. (2009). “Synthesis of azetidines,” in Science of Synthesis: Houben-Weyl Methods of Molecular Transformations, Vol. 40a, eds D. Enders and A. E. Schaumann (New York, NY: Georg Thieme, 773–816.

[B2] D'hooghe M. Ha H.-J (eds). (2016). Synthesis of 4- to 7-Membered Heterocycles by Ring Expansion: Aza-, Oxa-and Thiaheterocyclic Small-Ring Systems. Heidelberg: Springer.

[B3] Sweeney J. B. (2009). “Synthesis of aziridines,” in Science of Synthesis: Houben-Weyl Methods of Molecular Transformations, Vol. 40a, eds D. Enders and A. E. Schaumann (New York, NY: Georg Thieme, 643–772.

